# Human Dental Follicle Cell-Derived Small Extracellular Vesicles Attenuate Temporomandibular Joint Cartilage Damage through Inhibiting HIF-2*α*

**DOI:** 10.1155/2023/6625123

**Published:** 2023-09-25

**Authors:** Enyu Mao, Yu Hu, Yinzi Xin, Zheyi Sun, Jun Zhang, Song Li

**Affiliations:** ^1^Yunnan Key Laboratory of Stomatology, School of Stomatology, Kunming Medical University, Kunming, China; ^2^Department of Orthodontics, Kunming Medical University Affiliated Stomatological Hospital, Kunming, China; ^3^Kunming Medical University, Kunming, China

## Abstract

Mesenchymal stem cell (MSC)-based therapies for articular cartilage regeneration are effective mostly due to paracrine signals mediated by extracellular vesicles, especially small extracellular vesicles (sEV). However, it is unknown whether dental follicle cell-derived sEV (DFC-sEV) affect cartilage regeneration in temporomandibular joint osteoarthritis (TMJ-OA). In this study, the effects of DFC-sEV on IL-1*β*-induced mandibular condylar chondrocytes (MCCs) were determined using CCK8 assays, scratch assays, flow cytometry, and Western blot analysis of matrix synthesis and catabolic proteins. Furthermore, we used an abnormal occlusion-induced rat model and intra-articular injection of DFC-sEV, the pathological changes of which were observed by HE staining, safranin O staining, immunohistochemistry, and micro-CT analysis of subchondral bone loss. Gene set enrichment analysis (GSEA) was used to determine the related mechanism involved in the effect of DFC-sEV. Immunofluorescence analysis and Western blotting were used to evaluate the expression of HIF-1*α*, HIF-2*α*, MMP13, and VEGF in MCCs. Then, lentivirus-induced Epas1 overexpression and Western blot analysis of the downstream regulators of HIF-2*α* were performed. We found that DFC-sEV promoted MCCs proliferation and migration and protected against cartilage matrix destruction induced by IL-1*β*. In addition, DFC-sEV prevented cartilage destruction in an abnormal occlusion rat model. Furthermore, we found that DFC-sEV reduced the expression of HIF-1*α* and HIF-2*α* in vitro and in vivo and decreased the downstream regulators of HIF-2*α*, including MMP13 and VEGF. Our study indicated that DFC-sEV attenuated TMJ cartilage damage in vitro and in vivo, which might be involved in the regulation of HIF-2*α*.

## 1. Introduction

Temporomandibular joint osteoarthritis (TMJ-OA) is a progressive degenerative cartilage disease that has become more widespread and is increasingly being studied. Individuals suffering from TMJ-OA have joint pain and occlusion dysfunction, resulting in a decreased quality of life. The main characteristics of cartilage degradation in TMJ-OA include a reduction in the number of chondrocytes, loss of extracellular matrix (ECM), and subchondral bone modelling [1]. Cartilage damage occurs due to the abnormal status and function of chondrocytes, along with an imbalance in ECM formation and degradation [2]. The lack of blood vessels, nerves, and lymphoid tissue makes articular cartilage incapable of self-healing and regeneration [3]. Although traditional treatments have shown efficiency in pain relief, they are unable to reconstruct the cartilage [4, 5]. Mesenchymal stem cells (MSCs) can differentiate into osteoblasts and chondrocytes and have been shown to be effective in TMJ repair [6–8]; clinical applications have also been reported [9]. Notably, dental tissue-derived MSCs also exert therapeutic effects on TMJ-OA [10].

Recently, increasing evidence has suggested that MSCs secrete a wide range of soluble factors, microvesicles, and small extracellular vesicles (sEV) to regulate the tissue microenvironment and orchestrate regenerative processes [11]. Small EVs have membrane structures with diameters of 50–200 nm and contain proteins, lipids, and nucleic acids that can be transferred from donor cells to recipient cells and produce a therapeutic effect on OA [12–14]. MSC-derived sEV promote the repair and regeneration of cartilage in OA through different mechanisms, such as (1) enhancing matrix synthesis and preventing cartilage destruction; (2) promoting chondrocyte proliferation and migration while suppressing apoptosis; and (3) affecting immunomodulatory signals. However, most researchers have focused on the treatment of knee OA with MSC-derived sEV, and few studies have investigated TMJ-OA [15, 16]. The TMJ is a left-right linkage joint that is composed of fibrocartilage on the surface [17]; thus, the structural and functional features are different between the knee joint and TMJ. Dental stem cell-derived sEV have been shown to regulate many important biological processes, including inflammation, osteogenesis, angiogenesis, immunomodulation, and neuronal growth [18]. However, the therapeutic effects of dental follicle cell-derived sEV (DFC-sEV) on TMJ-OA have not been investigated.

TMJ cartilage is avascular and has a hypoxic environment. Hypoxia-inducible factor-2*α* (HIF-2*α*), which is encoded by Epas1, is markedly increased in osteoarthritic cartilage in vitro and in vivo [19–22]. HIF-2*α* is a catabolic factor that promotes the expression of matrix-degrading enzymes, including MMP9 and MMP13, and hypertrophic chondrocyte markers, such as COLX [23, 24]. Intra-articular delivery of anti-HIF-2*α* siRNA could prevent cartilage degeneration in osteoarthritic mice [25], and the downregulation of HIF-2*α* suppresses the development of OA [26–28]. However, whether sEV affect hypoxia-regulated proteins in TMJ-OA is unknown.

In this study, we evaluated the effects of DFC-sEV on mandibular condylar chondrocytes (MCCs) induced by IL-1*β* in vitro, and we performed intra-articular injections of DFC-sEV in a rat model of TMJ-OA. Then, we investigated cellular matrix homeostasis in TMJ cartilage regeneration. The actively expressed genes related to the therapeutic mechanism of DFC-sEV were explored through transcriptome sequencing. Finally, we concluded that DFC-sEV could attenuate TMJ cartilage damage and that DFC-sEV was associated with the regulation of HIF-2*α*.

## 2. Results

### 2.1. IL-1*β* Induced Inflammation and Injury in Rat MCCs (Figure 1)

**Figure 1:**
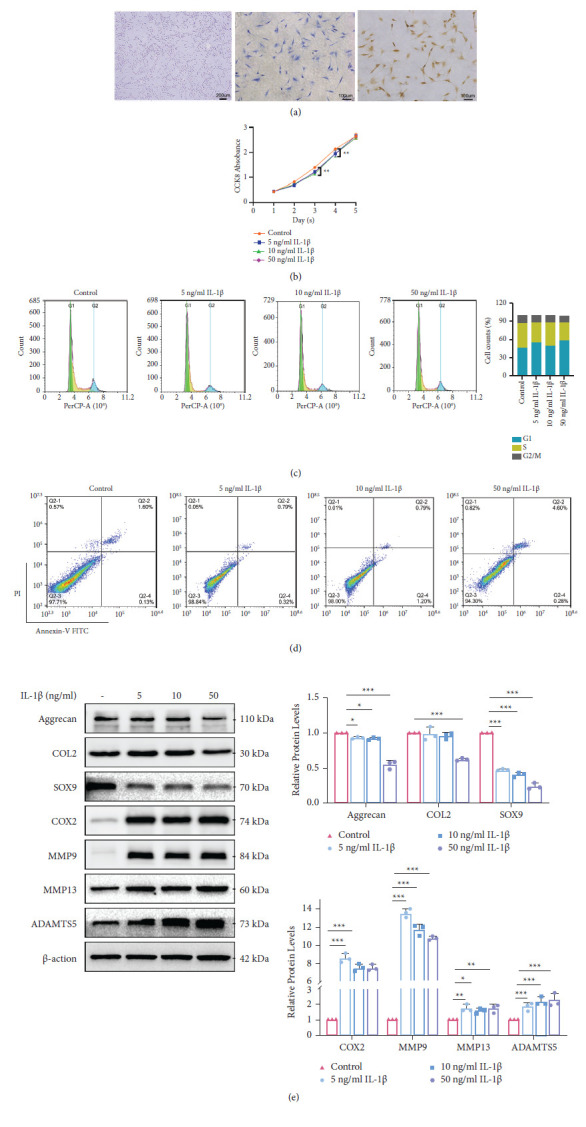
IL-I*β*-induced inflammation and injury in rat MCCs. (a) Morphology of MCCs in vitro and identification. (b) CCK8 assay detected the proliferation of MCCs with IL-I*β* (5, 10, 50 ng/ml). (c) IL-I*β* induced cell cycle arrest at the S and G2/M phases. (d) Flow cytometry analysis revealed that IL-I*β* significantly promoted cell apoptosis. (e) Expression of cartilage matrix-associated proteins, aggrecan, COL2, SOX9, COX2, MMP9, MMP13, and ADAMTS5. Data presented as mean ± SD, *n* = 3, ^*∗*^*P* < 0.05, ^*∗∗*^*P* < 0.01, ^*∗∗∗*^*P* < 0.001, and one-way ANOVA, followed by the Tukey' test.

MCCs were polygonal in shape and identified by toluidine blue and collagen II staining (Figure 1(a)). IL-1*β* was used to induce chondrocyte injury, and MCCs were stimulated with IL-1*β* for 24 h. The cell counting kit-8 (CCK8) assay revealed that IL-1*β* (5, 10, 50 ng/ml) dramatically inhibited the proliferation of MCCs (Figure 1(b)), and cell cycle analysis showed that IL-1*β* significantly reduced the percentage of MCCs in the S and G2 phases from 29.65% + 10.84% to 39.47% + 13.24%, respectively, in response to 50 ng/ml IL-1*β* (Figure 1(c)). These results suggest that IL-1*β* induces cell cycle arrest and may inhibit proliferation. Furthermore, flow cytometry indicated that the proportion of early apoptotic cells increased in response to 50 ng/ml IL-1*β* from 1.6% to 4.6%, but this effect was not obvious at the other concentrations (Figure 1(d)). Western blot analysis revealed that IL-1*β* significantly inhibited the expressions of aggrecan, COL2, and SOX9 while promoted COX2, MMP9, MMP13, and ADAMTS5 expressions (Figure 1(e)). The results showed that 10 ng/ml IL-1*β* induced mandibular condylar chondrocytes injury and inflammation, so it was used for subsequent experiments.

### 2.2. DFC-sEV Promoted MCCs Proliferation and Migration, Inhibited MCCs Apoptosis, and Protected against Cartilage Matrix Destruction Induced by IL-1*β* (Figure 2)

**Figure 2:**
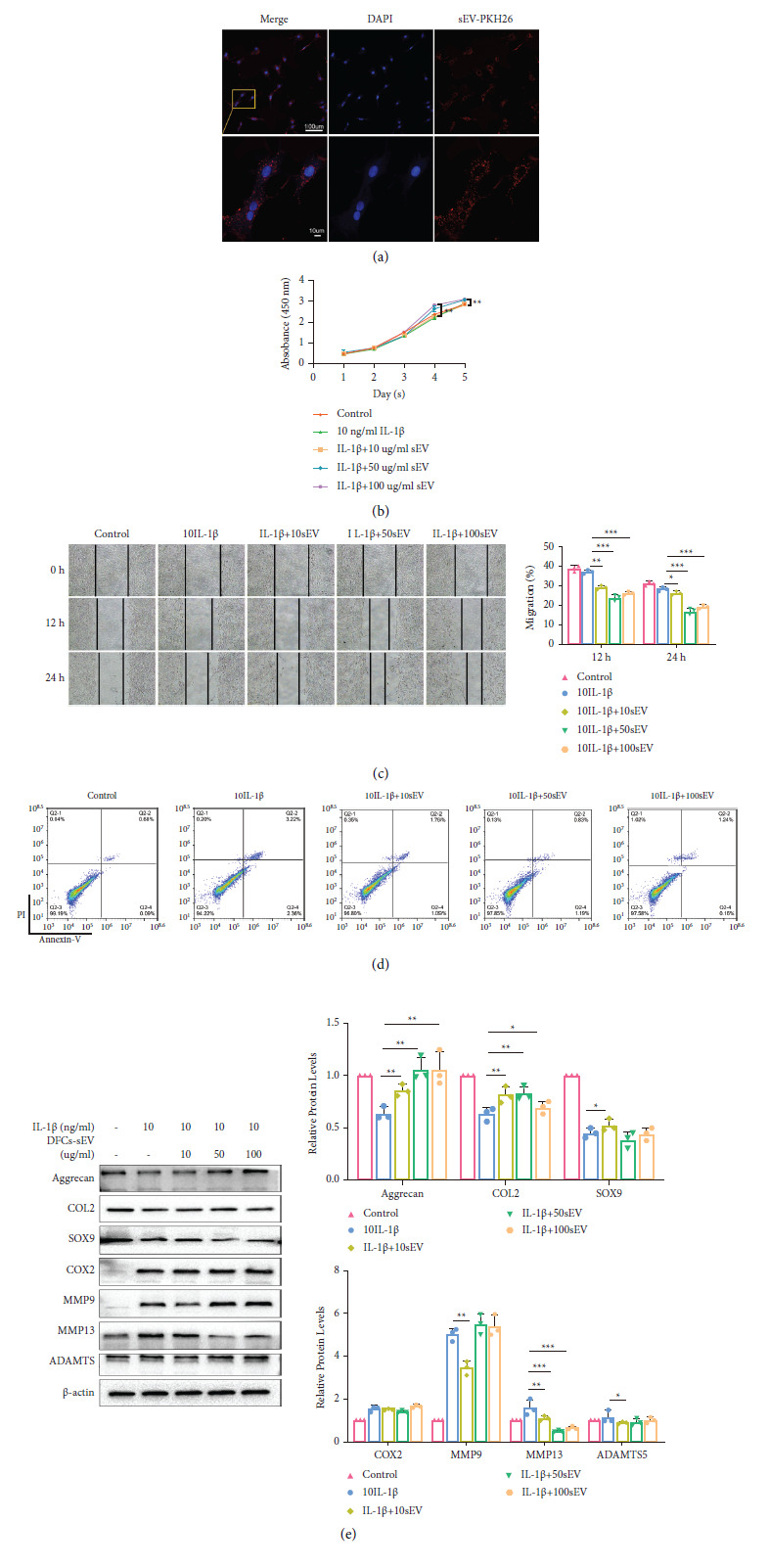
DFC-sEV promoted proliferation and migration, inhibited apoptosis, and protected against cartilage matrix destruction in MCC inducted by IL-I*β*. (a) DFC-sEV internalization by chondrocyte. The nucleus of MCCs was stained with DAPI (blue), and the sEV were labelled with PKH26 (red). Scale bar: 100 *μ*m and 10 *μ*m (magnified image). (b) The CCK8 assay showed that DFC-sEV promoted MCC proliferation under inflammatory conditions. (c) DFC-sEV promoted MCC migration under inflammatory conditions in a scratch-wound healing assay, scale bar: 200 *μ*m. (d) DFC-sEV inhibited IL-I*β*-induced apoptosis in MCC. (e) Expression of the cartilage matrix-associated proteins aggrecan, COL2, SOX9, COX2, MMP9, MMP13, and ADAMTS5. Data presented as the mean ± SD, *n* = 3, ^*∗*^*P* < 0.05, ^*∗∗*^*P* < 0.01, ^*∗∗∗*^*P* < 0.001, and one-way ANOVA, followed by the Tukey' test.

After MCCs were treated with DFC-sEV for 12 h, PKH26-labelled DFC-sEV (red fluorescence) was internalized by MCCs (Figure 2(a)). To investigate the effect of DFC-sEV on TMJ-OA, MCCs were cotreated with IL-1*β* and DFC-sEV for 24 h. CCK8 and scratch-wound healing assays revealed that DFC-sEV promoted the proliferation and migration of MCCs in vitro; moreover, in the 50 *µ*g/ml DFC-sEV and 100 *µ*g/ml DFC-sEV groups, the effect was more pronounced (Figures 2(b) and 2(c)). The flow cytometry showed that the proportions of early and late apoptotic cells significantly decreased in response to IL-1*β* and DFC-sEV in the 10, 50, and 100 *µ*g/ml DFC-sEV groups, especially in the 50 *µ*g/ml DFC-sEV group, and early apoptotic cells were reduced from 3.22% to 0.83% (Figure 2(d)). Western blot analysis revealed that DFC-sEV promoted the expression of cartilage matrix synthesis proteins (Aggrecan, COL2, and SOX9) to varying degrees; moreover, DFC-sEV inhibited the expression of catabolic proteins (MMP9, MMP13, and ADAMTS5) but had no effect on COX2. Notably, protein expression did not have a linear relationship with the DFC-sEV concentration (Figure 2(e)).

### 2.3. DFC-sEV Prevented Cartilage Destruction in an Abnormal Occlusion Rat Model (Figure 3)

**Figure 3:**
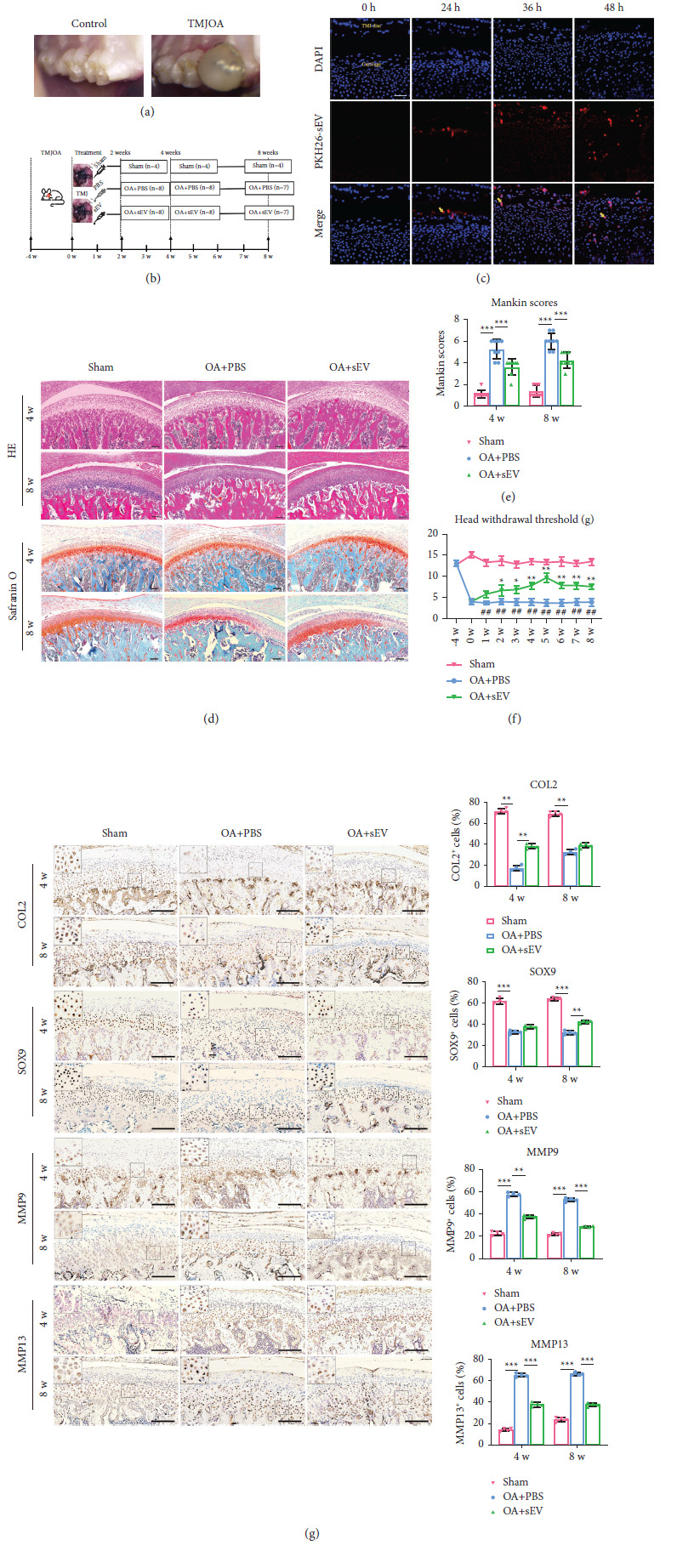
DFC-sEV prevents cartilage destruction in an abnormal occlusion rat model. (a) Representative image of the first molar occlusion relationship between the control and TMJOA groups. (b) Schematic diagram of the experimental schedule and grouping design. (c) Representative image of PKH26-labelled DFC-sEV in the TMJ after sEV injection into the cavity joint. (d, e) HE and safranin O/fast green staining of condylar cartilage at week 4 and week 8 and Mankin scores among different groups, *n* = 8, per group. Scale bar: 100 *μ*m, ^*∗*^*P* < 0.05, ^*∗∗*^*P* < 0.01, ^*∗∗∗*^*P* < 0.001, and one-way ANOVA, followed by the Tukey' test. (f) Time-dependent nociceptive responses following treatment with DFC-sEV, ^*∗*^*P* < 0.05, ^*∗∗*^*P* < 0.01 compared to OA + PBS group; ^#^*P* < 0.05, ^##^*P* < 0.01, compared to sham group. (g) Immunohistochemistry analysis of Co12, SOX9, COX2, MMP9, and MMP13 in the cartilage layer, *n* = 4 per group, scale bar; 100 *μ*m, ^*∗*^*P* < 0.05, ^*∗∗*^*P* < 0.01, ^*∗∗∗*^*P* < 0.001, and one-way ANOVA, followed by the Tukey' test.

To evaluate the role of DFC-sEV in the protection of condylar cartilage in vivo, we used an abnormal occlusion rat model of TMJ-OA (Figure 3(a)). In this study, treatment was started 4 weeks after modelling. Intra-articular administration of DFC-sEV or PBS was performed on a weekly basis for 2, 4, and 8 weeks (Figure 3(b)). First, PKH26-labelled sEV (PKH26-sEV) were injected into the joint in rats with abnormal occlusion to determine whether the sEV could enter the destructive articular cartilage. PKH26-sEV were detected 24 h after intra-articular injection. Over time, the PKH26-sEV gradually moved into the cartilage from the exterior to the interior, and PKH26-sEV could even be observed in the hypertrophic layer (Figure 3(c)).

HE and safranin O fast green staining showed OA-like changes that were characterized by reduced cartilage thickness, the loss of chondrocytes, and a less ordered arrangement of cartilage, as well as the extensive loss of proteoglycans after 4 weeks of abnormal occlusion in rats. Over time, these changes became more obvious. After 4 and 8 weeks of DFC-sEV treatment, increased numbers of chondrocytes and restored proteoglycan deposition were observed in the OA + sEV group, and the cartilage had different degrees of thickening (Figure 3(d)). The Mankin scores, including the maximal and summed scores, were used to quantify the severity of cartilage damage; the results revealed that cartilage lesions were significantly impaired after abnormal occlusion modelling in rats, while sEV could reduce cartilage injury (4 w, *F* = 15.278, *P* < 0.0001; 8 w, *F* = 16.125, *P* < 0.0001) (Figure 3(e)). At the same time, OA + sEV rats had reduced pain as indicated by higher HWT than those in the OA + PBS group as early as 2 weeks after treatment (*P*=0.025). The pain was reduced to baseline and was similar to that of the sham group. This was then maintained for the duration of the treatment. In contrast, the OA + PBS group showed minimal HWT improvement throughout the course of the experiment (Figure 3(f)).

Moreover, immunohistochemical experiments were performed to assess the changes in cartilage matrix protein levels in rat cartilage. As shown in Figure 3(g), in the OA group, the cartilage matrix consisting of COL2 and SOX9 in cartilage was obviously decreased (COL2, 4 w, *F* = 9.518, *P*=0.0049) (SOX9, 8 w, *F* = 8.156, *P*=0.0054), while the expressions of MMP9 (4 w, *P*=0.0015; 8 w, *P* < 0.0001) and MMP13 (4 w, *P* < 0.0001; 8 w, *P* < 0.0001) were upregulated. In the OA + sEV group, cartilage matrix loss was less severe compared to the OA group; the expressions of COL2 and SOX9 were significantly increased. Collectively, these results revealed that DFC-sEV could enter the damaged articular cartilage, promote chondrocyte catabolism, and inhibit anabolism, which prevented cartilage destruction in TMJ-OA.

### 2.4. DFC-sEV Improved Subchondral Bone Structure (Figure 4)

**Figure 4:**
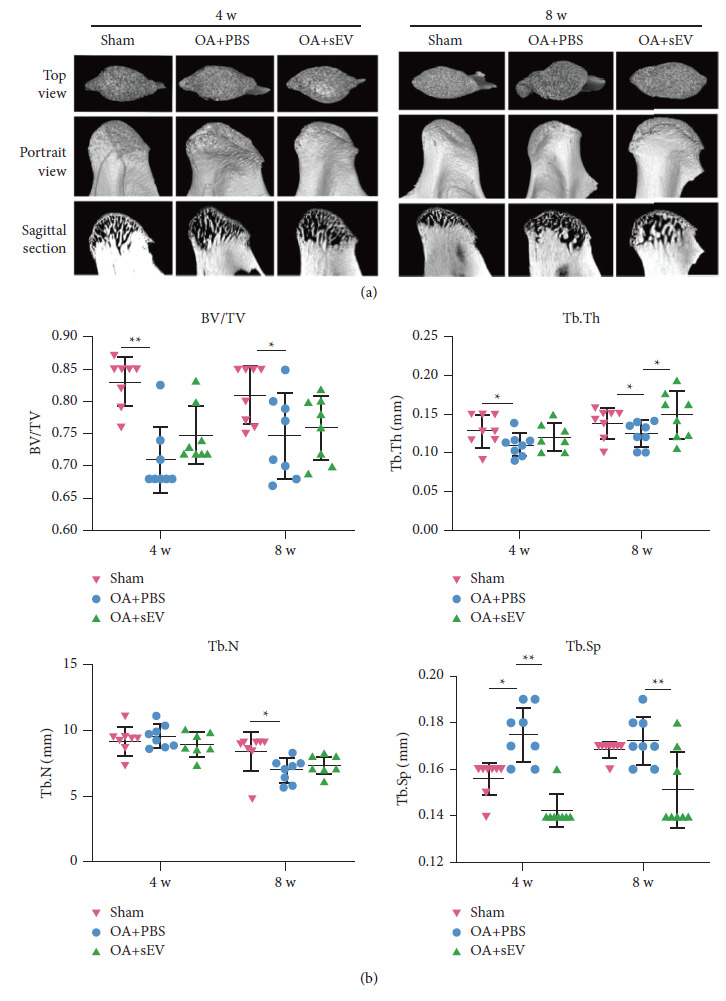
DFC-sEV improve the subchondral bone structure. (a) Representative image of the top view, portrait view, and sagittal section of TMJ condyle at week 4 and week 8. (b) Bone loss parameters: ratios of bone volume (BV/TV), trabecular thickness (Tb, Th), trabecular number (Tb.N) and trabecular separation (Tb.Sp), *n* = 8 per group, ^*∗*^*P* < 0.05, ^*∗∗*^*P* < 0.01, ^*∗∗∗*^*P* < 0.001, and one-way ANOVA followed by the Tukey' test.

Our previous results demonstrated that DFC-sEV have therapeutic effects on cartilage, and we next investigated the changes in the subchondral bone. The three-dimensional reconstructed images showed that rats in the OA + PBS group developed trabecular bone loss compared to rats in the sham group after 4 weeks of modelling, as follows: the ratio of bone volume to tissue volume (BV/TV) and trabecular thickness (Tb.Th) decreased, trabecular separation (Tb.Sp) increased, and there was no difference in trabecular number (Tb.N) (Figures 4(a) and 4(b)). Significant differences in four bone structural parameters were observed at week 8, and there was a significant variation between the OA + PBS and sham groups. Moreover, compared to the OA + PBS group, the OA + sEV group had a decrease in Tb.Sp at week 4 (*F* = 12.579, *P*=0.039) and week 8 (*F* = 8.375, *P*=0.057), and with time, Tb.Th increased at week 8 (*F* = 9.369, *P*=0.0028). These results indicated that DFC-sEV could improve trabecular bone mass by enhancing bone formation and inhibiting bone resorption.

### 2.5. Differences in Transcriptome Signatures before and after DFC-sEV Treatment (Figure 5)

**Figure 5:**
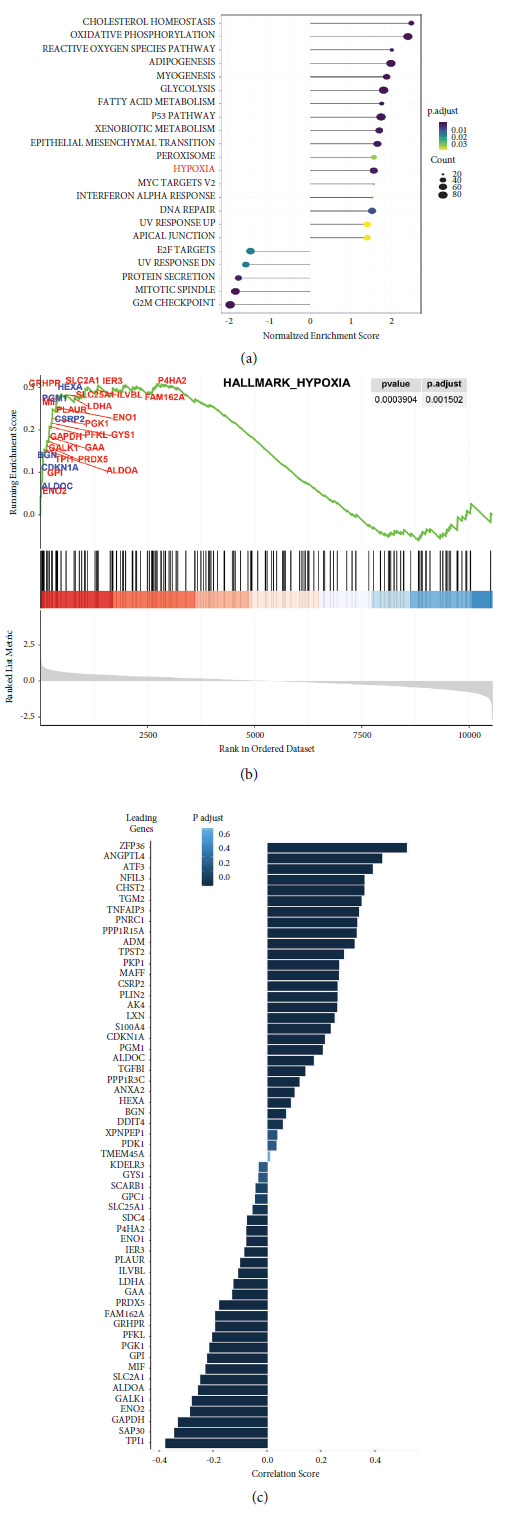
Differences in transcriptome signatures before and after DFC-sEV treatment. (a) GSEA of the hypoxia signalling pathway. (b) The leading-edge subset genes positively (blue) and negatively (red) related to hypoxia function. (c) Correlation analysis between leading-edge genes and HIF-2*α*.

To explore the mechanism by which DFC-sEV prevent cartilage destruction in TMJ-OA, we extracted the total RNA from each group in vitro cell culture (control, 10IL-1*β* and 10IL-1*β* + 50sEV) for transcriptomic sequencing. Gene set enrichment analysis (GSEA) suggested that the hypoxia signalling pathway was activated by DFC-sEV treatment (Figure 5(a)), and the GSEA plot showed representative genes in the leading edge that were positively (blue) and negatively (red) related to the hypoxia pathway (Figure 5(b)). These results suggested that in the leading-edge subset genes, the genes that were negatively related to the hypoxia pathway played a dominant role and were highly correlated with HIF-2*α* (Figure 5(c)).

### 2.6. DFC-sEV Inhibited the Expression of HIF-1*α* and HIF-2*α* (Figure 6)

**Figure 6:**
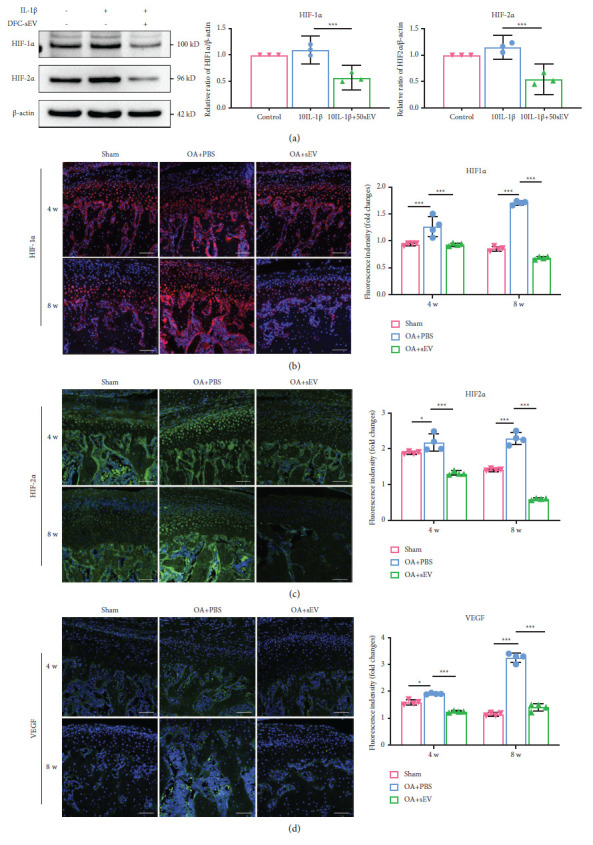
DFC-sEV inhibits the expression of HIF-1*α* and HIF-2*α*. (a) Western blot analysis of HIF-1*α* in MCCs treated with IL-I*β* and DFC-sEV, *n* = 3, ^*∗*^*P* < 0.05, ^*∗∗*^*P* < 0.01, ^*∗∗∗*^*P* < 0.001, one-way ANOVA followed by the Tukey' test. (b–d) Immunofluorescence staining image of HIF-1*α* (b), HIF-2*α* (c), and VEGF (d) in condylar cartilage and subcondral bone, *n* = 4, scale bar: 100 *μ*m, ^*∗*^*P* < 0.05, ^*∗∗*^*P* < 0.01, ^*∗∗∗*^*P* < 0.001, and one-way ANOVA followed by the Tukey' test.

HIF-1*α* and HIF-2*α* were shown to be involved in hypoxia progression in OA; thus, we hypothesized that HIF-1*α* and HIF-2*α* may contribute to the therapeutic effect of DFC-sEV on TMJ-OA. First, we validated the changes in HIF-1*α* and HIF-2*α* after DFC-sEV treatment. Western blot analysis showed that the expression of HIF-1*α* and HIF-2*α* increased in the presence of IL-1*β*, and this effect was reversed by DFC-sEV (*P* < 0.0001) (Figure 6(a)).

Then, we examined the changes in vivo by immunofluorescence staining. The results revealed that HIF-1*α* (Figure 6(b)) and HIF-2*α* (Figure 6(c)) fluorescence could be observed in the superficial, hypertrophical, and subchondral bone zones in the condyle, while VEGF (Figure 6(d)) was observed in the subchondral bone zone, especially in the marrow cavity. After 4 and 8 weeks of DFC-sEV treatment, the levels of HIF-1*α*, HIF-2*α*, and VEGF decreased compared with those in the OA + PBS group (*P* < 0.001).

### 2.7. The Prevention of Cartilage Destruction by DFC-sEV Was Mediated by HIF-2*α* (Figure 7)

**Figure 7:**
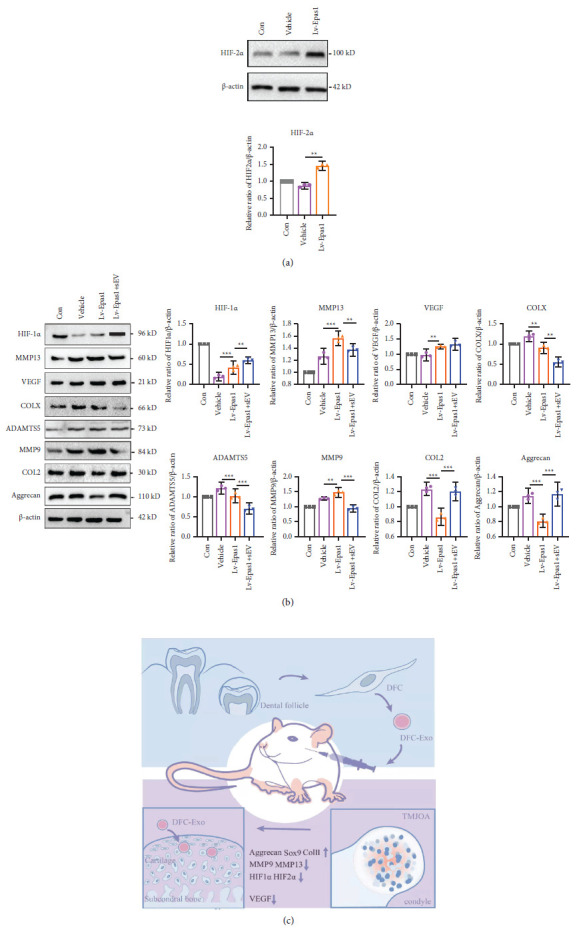
HIF-2*α* is involved in the prevention of cartilage destruction with DFC-sEV. (a) Western blot analysis of HIF-2*α* after overexpression of Epas-1 in normal MCC. (b) Western blot analysis of HIF-1*α*, MMP13, VECF, COLX, and cartilage matrix associated proteins after overexpression of Epas-1 in normal MCC, *n* = 3, ^*∗*^*P* < 0.05, ^*∗∗*^*P* < 0.01, ^*∗∗∗*^*P* < 0.001, and one-way ANOVA followed by the Tukey' test. (c) A proposed mechanism of DFC-sEV in protecting against matrix destruction in TMJ-OA.

To further confirm whether HIF-2*α* is involved in DFC-sEV-mediated prevention of cartilage destruction, we performed an in vitro rescue experiment. We overexpressed the Epas-1 gene in normal MCCs (Figure 7(a)) and found that the protein levels of HIF-1*α*, MMP13, and VEGF were also increased. Furthermore, after Epas-1 was successfully overexpressed, MCCs were treated with 50 *µ*g/ml DFC-sEV. The results showed that MMP13, COLX, MMP9, and ADAMTS5 were significantly decreased after DFC-sEV treatment (MMP13, *F* = 6.357, *P*=0085; COLX, *F* = 8.236, *P*=0.0073; MMP9, *F* = 10.287, *P* < 0.0001; ADAMTS5, *F* = 16.378, *P* < 0.0001), while HIF-1*α*, COL2, and Aggrecan were increased (HIF-1*α*, *F* = 8.359, *P*=0.0075; COL2, *F* = 9.127, *P* < 0.0001; ADAMTS5, *F* = 13.287, *P* < 0.0001); there was no change in VEGF expression (*P* > 0.05) (Figure 7(b)).

## 3. Materials and Methods

### 3.1. Extraction and Identification of DFC-sEV

When the cells reached 80% confluence, the medium was changed to MSC serum-free medium (SFM, Thermo Fisher Scientific), and the cells were incubated for 48 h. Then, the supernatant was collected and concentrated with a 3 kDa ultrafiltration tube (Millipore) at 5,000 × g for 30 min at 4°C. sEV were extracted from the concentrated supernatant with the Exo Easy Maxi Kit (Qiagen) as previously reported [29]. The concentration of DFC-sEV was measured by a BCA Protein Assay Kit (Beyotime).

Transmission electron microscopy (TEM) was used to observe the morphology of sEV. Nanoparticle tracking analysis (NTA) was used to determine the diameters and concentrations of sEV. sEV surface markers, including CD9, CD81, HSP90, and TSG101, were analysed by Western blotting.

### 3.2. Isolation and Culture of MCCs

Condylar cartilage was dissected from 3–5 days old male Sprague-Dawley rats. The tissues were digested with 0.2% collagenase II for 30 min. Then, the digested tissues were placed in high-glucose medium (BI) containing 10% FBS (Gibco) and 1% penicillin‒streptomycin (HyClone) at 37°C and 5% CO_2._ Second- or third-passage cells were used for experiments.

### 3.3. Cellular Internalization of DFC-sEV

Approximately, 10 *µ*g of DFC-sEV was labelled with 6 *µ*l of PKH26 dye (Sigma-Aldrich) in 1 ml of diluent C. Then, the labelled DFC-sEV were concentrated in ultrafiltration tubes at 4°C and 5,000 × g for 30 min. After being incubated with labelled DFC-sEV for 8 h, MCCs were washed with PBS, fixed with 4% paraformaldehyde, stained with DAPI, and then observed under a laser scanning confocal microscope (Nikon).

### 3.4. IL-1*β*-Induced MCCs Inflammation and DFC-sEV Treatment

MCCs were stimulated with 5, 10, and 50 ng/ml IL-1*β* (Origene) for 24 h to induce osteoarthritic inflammation as previously described, and a final concentration of 10 ng/ml was used for subsequent experiments. To evaluate the protective effects of DFC-sEV against TMJ-OA, IL-1*β* was added, and different concentrations (10, 50, and 100 *µ*g/ml) of DFC-sEV were also added to the medium and incubated for 48 h. Subsequent in vitro experiments were performed with 50 *µ*g/ml DFC-sEV.

### 3.5. Proliferation Assay

At 24, 48, 72, and 96 h after treatment with IL-1*β* and DFC-sEV, a CCK8 assay (MeilunBio) was used to measure the absorbance at 450 nm with a microplate reader (Thermo). Cell cycle analysis (MeilunBio) was performed according to the manufacturer's instructions. The stained cells were analysed on an Agilent NovoCyte Fluidics Station (Agilent Technologies, Santa Clara) 48 h after treatment.

### 3.6. Flow Cytometric Analysis of Apoptosis

Cell apoptosis was analysed with an Annexin V-FITC/PI Apoptosis Detection Kit (MeilunBio). Cells were collected after 48 h of treatment, and then 500 *μ*l of 1x binding buffer was used to resuspend the cells after centrifugation. Finally, 5 *μ*l of Annexin V-FITC and 5 *μ*l of PI were added to 100 *μ*l of the cell suspension, and the samples were shielded from light and incubated for 15 min for flow cytometric analysis.

### 3.7. Scratch Assay

MCCs were seeded into a 24-well plate at a density of 1 × 10^5^ cells/well with a Culture-Insert (Ibidi) placed at the centre of each well. Scratches with a width of 500 *μ*m were formed by removing the insert the next day. After treatment, the same position was photographed using an inverted microscope (Olympus CKX53).

### 3.8. Western Blot Analysis

Western blotting was performed according to the study as previously described. Briefly, the proteins were loaded onto SDS-PAGE gels and electroblotted onto PVDF membranes (Millipore). Antibodies against Aggrecan (ab3686), COL2 (ab188570), SOX9 (82630T, CST), COX2 (12282T, CST), MMP9 (13667T, CST), MMP13 (69926S, CST), ADAMTS5 (ab41037), and *β*-actin (TA811000, Origene) were used. The protein bands were examined with a Bio-Rad chemiluminescence imaging system.

### 3.9. Animal Experimental Protocols

All procedures were approved and conducted in accordance with the guidelines of the Animal Experiment Ethics Review Committee of Kunming Medical University (approval no. KMMU20211594). A total of 80 six-week-old male SD rats (weighing 160–180 g) were used in this study. The animals were randomly divided into three groups as follows: sham (*n* = 14), OA + PBS (*n* = 26), and OA + sEV (*n* = 26) (Figure 4(b)). TMJ-OA was induced by abnormal occlusion as previously reported [30]. Briefly, a 0.25 mm orthodontic ligation silk was inserted between the first and second molars, and then, the silk knot containing fluid resin (3 M) at the first molar of the maxilla induced abnormal mechanical loading on the TMJ. Then, the rats were administered weekly intra-articular injections of 50 *μ*l of 1 mg/ml DFC-sEV in the OA + sEV group or 50 *μ*l of PBS in the OA + PBS group twice per week for 4 and 8 weeks. The sham group was only subjected to ligation silk inserted between the first and second molars. All animals were allowed free access to water and food and were kept in a controlled environment (50% humidity, 25°C, and 12 h light-dark cycle).

### 3.10. Histologic Analysis and Immunohistochemistry

The rats were euthanized by a fivefold overdose of 3% sodium pentobarbital, and TMJ tissues were harvested. The TMJ samples were fixed in 4% paraformaldehyde for 24 h. After being decalcified in 10% EDTA, the samples were embedded in paraffin and cut into 5 *μ*m sagittal sections. HE and safranin O fast green staining were used to analyse the changes in the cartilage structure and proteoglycans. The histomorphology grade was determined with modified Mankin scores [31], and the scores represented the severity of the injury.

Immunohistochemistry and immunofluorescence analyses were performed as previously described. Briefly, 1% sodium citrate was used for antigen retrieval, and endogenous peroxidase activity was quenched with 3% H_2_O_2_. The sections were incubated overnight at 4°C with antibodies against COL2 (ab188570), SOX9 (82630T, CST), MMP9 (13667T, CST), and MMP13 (69926S, CST) for immunohistochemistry and HIF-1*α* (Invitrogen, PA1-16601), HIF-2*α* (Genespan, GXP229610), and VEGF (Invitrogen) for immunofluorescence analysis. Three fields per section were selected randomly for statistical analysis. The total number of chondrocytes and those that stained positive in three central regions of articular cartilage were counted using Image-Pro Plus version 6.0 software. Positive fluorescence signals were quantified with ImageJ software.

### 3.11. Pain Behavioral Measurement

Mechanical hypernociception was assessed using the von Frey microfilament procedure under blind conditions. The rats were tested with a series of filaments (Aesthesio, Damnic global, CA, USA) at the preauricular area. The head withdrawal threshold (HWT) is defined as the lowest force of the filaments that produced the withdrawal response for at least three times. The assessment of mechanical hypernociception was performed at weekly intervals and the HWT was calculated as a mean value per joint of 4 rats per group.

### 3.12. Micro-CT Analysis

Before decalcification, a NEMOR NMC-100 micro-CT system (PINGSENG Healthcare) was used to scan the subchondral bone with the following parameters: 90 kV source voltage and 60 *μ*A source current. When the top and portrait views were reconstructed, BV/TV, Tb.Th (mm), Tb.N (mm), and Tb.Sp (mm) were measured by Avatar 1.5.0 software (PINGSENG Healthcare).

### 3.13. Functional Enrichment Analysis by GSEA

We treated the cells with DFC-sEV under IL-1*β*-induced inflammatory conditions, and Novogene (China) performed a 150-bp paired-end RNA-seq experiment on an Illumina HiSeq 3000 platform with two biological replicates in each group. We used the DESeq2 R package for differential analysis and the cluster Profiler [32] R package for GSEA in which the hallmark gene set was used. Correlation analysis of HIF2*α* with all other hypoxia-enriched genes identified by GSEA was performed on the TCGA-BRCA (phs000178) dataset. All data visualization was powered by R studio with R version 4.2.0.

### 3.14. MCCs Overexpressing *Epas1* by Lentiviral Transfection

Transfections with luciferase were performed when MCCs reached 30–40% confluence. The transfection reagent was purchased from Obio Technology (Shanghai, China), and the following MOIs were used: Lv-Epas1 (100 MOI) and Lv-vehicle (80 MOI). Green fluorescence was observed 48 h after transfection, and positive cells were selected using puromycin. Then, 50 *µ*g/ml DFC-sEV was added to the culture and incubated for 48 h. Finally, the total protein extract was prepared for Western blotting as previously described, and the antibodies used were anti-VEGF (Santa Cruz Biotechnology, sc-7269) and anti-COLX (GTX37732).

### 3.15. Statistical Analysis

All experimental data were statistically analysed using SPSS 19.0 software. The data are expressed as the mean ± standard deviation and one-way ANOVA, followed by Tukey's test which was used for multiple comparisons. A value of *P* < 0.05 was considered to be statistically significant (^*∗*^*P* < 0.05, ^*∗∗*^*P* < 0.01, ^*∗∗∗*^*P* < 0.001).

## 4. Discussion

Although many studies have demonstrated that MSC-derived sEV are effective in the repair and regeneration of articular cartilage in the knee OA [33–36], research on the therapeutic effects on TMJ-OA is lacking [15], and there have been no reports about treating TMJ-OA with dental tissue-derived sEV. DFCs originate from the neural crest, and these cells can differentiate into chondrocytes and osteoblasts [37]. DFCs are derived from tissues at an early stage of tooth development, and isolation is harmless and minimally invasive; moreover, studies have shown that DFCs have a higher proliferative ability than other dental stem cells [38]. Studies have increasingly demonstrated that MSC-based therapies are effective due to sEV-mediated paracrine secretion [12], and so we hypothesized that DFC-sEV may have therapeutic effects on TMJ-OA.

In this study, we demonstrated that DFC-sEV were internalized by chondrocytes, promoted proliferation and migration, inhibited apoptosis, and protected against cartilage matrix destruction in MCCs exposed to IL-1*β* in vitro, which was consistent with previous studies in OA [39–41]. IL-1*β* is one of the most significant mediators of cartilage degeneration and inflammation, and it can also stimulate chondrocytes to produce catabolic enzymes such as MMP9, MMP13, and ADAMTS5 [42, 43]. In addition, the concentrations of IL-1*β* used differed in other studies and cells, and so we first verified the effects of different concentrations of IL-1*β*, and 10 ng/ml was determined to be an appropriate concentration, which is consistent with other studies [44–46]. Our results indicated that DFC-sEV promoted chondrocyte proliferation, inhibited chondrocyte apoptosis, and protected the balance between cartilage construction and destruction [47]. Notably, different concentrations of DFC-sEV had different time-dependent effects, which can be explained by the different emergence times of corresponding proteins, and it takes time for sEV to exert their effects.

In addition, we performed animal experiments to verify the therapeutic effects of DFC-sEV in vivo. An increasing number of studies have shown that aberrant mechanical loading on the TMJ is one of the main factors in TMJ-OA [1, 48], so we established a rat model by inducing abnormal occlusion. Before treatment, PKH26-sEV were injected into the joint to observe whether cartilage tissues could take up DFC-sEV. We found that DFC-sEV could reach the superficial cartilage and joint cavity 24 h after injection, and with time, they were observed in the hypertrophic layer at 48 h. This result suggested that DFC-sEV could be delivered from the exterior to the interior of the cartilage, and during this process, sEV made a difference. Histological analysis showed that after 4 weeks and 8 weeks of treatment, the OA + sEV group exhibited marked restoration of cartilage structure and cellular matrix deposition compared with that in the sham group. Moreover, intra-articular injection of DFC-sEV decreased the expression of MMP9 and MMP13 and increased the expression of COL2 and SOX9. These findings were similar to the findings of other studies on OA [49–52]. In summary, we confirmed that DFC-sEV promoted the MCC proliferation and migration and inhibited apoptosis and that DFC-sEV could be delivered into the deeper layer of cartilage. The sEV may be absorbed by chondrocytes and play a role in protecting against cartilage matrix destruction.

To elucidate the mechanism underlying the effects of DFC-sEV on the synthesis and degradation of cartilage matrix, we analysed the mRNA profiles of IL-1*β*-induced inflammatory MCCs before and after DFC-sEV treatment. GSEA demonstrated that genes related to the hypoxia pathway might be activated, and among the leading-edge subset genes, the genes that were negatively related to hypoxia had a dominant role, suggesting that the expression of hypoxia-responsive genes, including HIF-1*α* and HIF-2*α*, may be reduced. To further validate this hypothesis, we conducted additional experiments.

Chondrocytes are particularly susceptible to low oxygen pressure, and hypoxia is considered an important factor for cartilage matrix synthesis and degradation [53, 54]. Our previous study showed that HIF-2*α* was highly expressed in condylar cartilage in an abnormal occlusion rat model [55], and this phenomenon was observed in osteoarthritic surgical specimens [20, 21]. HIF-2*α* regulates the expression of catabolic factors, including MMP3, MMP9, MMP13, and ADAMTS4 [56]. In addition, COLX, MMP13, and VEGFA are direct transcriptional targets of HIF-2*α* that stimulate endochondral ossification [21]. Intra-articular injection of nanoparticles containing HIF-2*α* siRNA could specifically inhibit the expression of catabolic proteins [25].

Although the protective effects of HIF-1*α* on OA have been documented, the oxygen concentration and duration of exposure affect its expression [57]. Our results showed that the expressions of HIF-1*α* and HIF-2*α* were increased in the IL-1*β*-induced inflammation model in vitro and the abnormal occlusion model in rats, and DFC-sEV reduced their expression. The changes in HIF-1*α* were similar to those in other studies of OA [57–59], while in some studies, the expression of HIF-1*α* was increased by damage [60]. The reasons for these differences are due to the different cell types and different time points used for detection. In addition, after DFC-sEV treatment, the downstream regulators of HIF-2*α*, including MMP13 and VEGF, were decreased. Then, we confirmed that DFC-sEV could inhibit the expression of downstream regulators caused by HIF-2*α* overexpression. However, how HIF-2*α* is involved in the therapeutic effects remains to be addressed.

## 5. Conclusions

The main characteristics of cartilage degradation in TMJ-OA include reduced number of chondrocytes, the loss of ECM, and subchondral bone remodelling. We found that DFC-sEV promoted MCC proliferation and migration, inhibited apoptosis, and protected against cartilage matrix destruction induced by IL-1*β*. Moreover, DFC-sEV could be delivered to the deeper layer of cartilage and prevent cartilage destruction in an abnormal occlusion rat model. Furthermore, we identified that the therapeutic effect involved the regulation of HIF-2*α*. This study supports a novel therapeutic strategy for TMJ-OA.

## Data Availability

All data generated or analysed during this study are included in this article and its supplementary materials.

## Supplementary Materials

Supplementary Materials Figure S1: Characterization of DFCs and DFC-sEV. (A) The morphology of DFCs, cells formed colonies, and differentiation into adipocytes and osteoblasts in vitro. (B) Flow cytometric analysis of MSCs surface markers. (C) Morphological features of DFC-sEV were observed via transmission electron microscopy, scale bar = 200 nm. (D) DFC-sEV size and concentration were monitored with NTA. (E) Western blot analysis shows the expression of the representative markers CD9, CD81, HSP90, and TSG101.
