# Supplementary figures and images for: Human Dental Follicle Cell-Derived Small Extracellular Vesicles Attenuate Temporomandibular Joint Cartilage Damage through Inhibiting HIF-2α
Source: J Tissue Eng Regen Med. 2023 Sep 25;2023:6625123. doi: 10.1155/2023/6625123 (PMC11919129; doi:10.1155/2023/6625123)

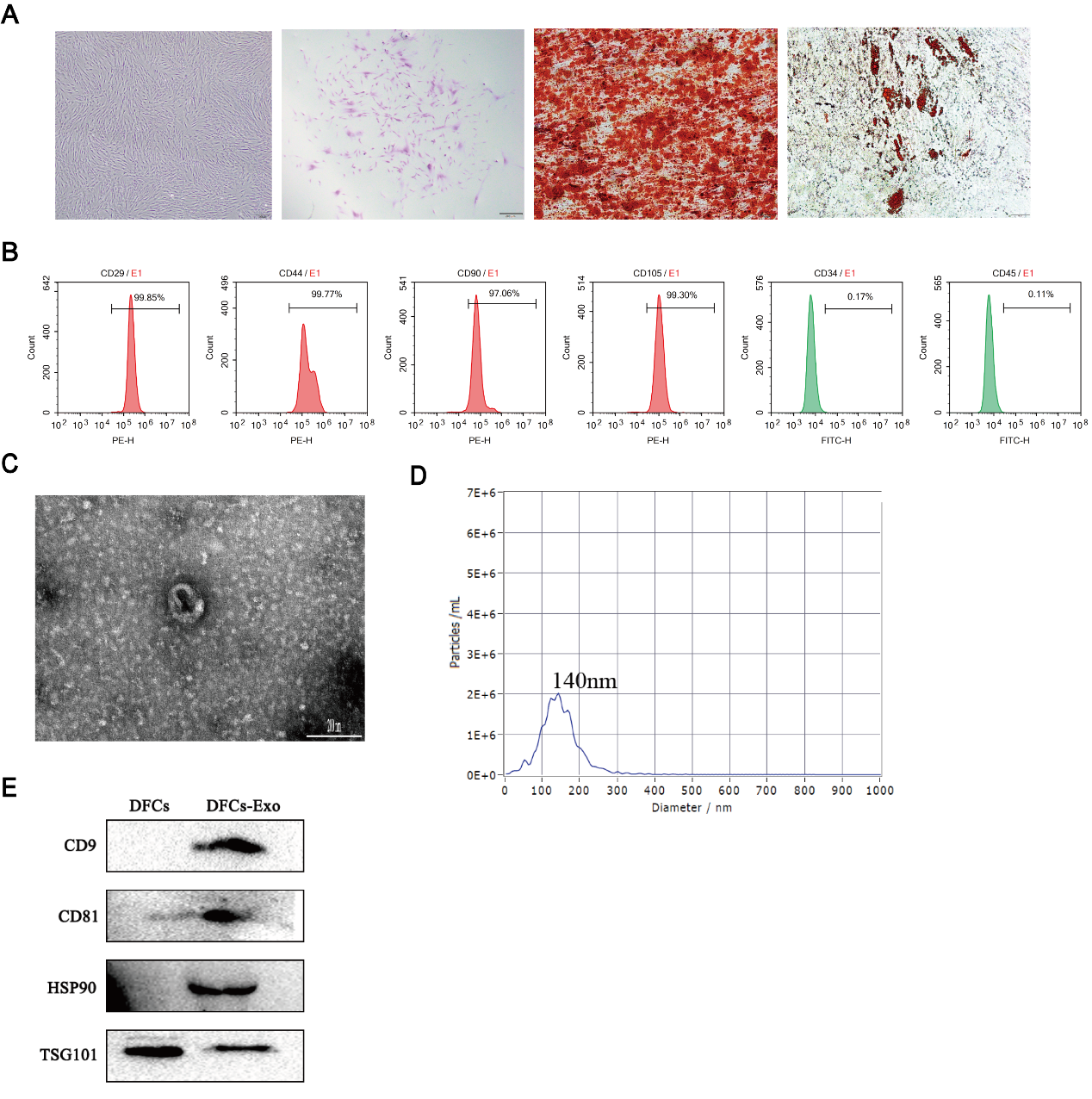

Supplement: Supplementary Materials — Figure S1: Characterization of DFCs and DFC-sEV. (A) The morphology of DFCs, cells formed colonies, and differentiation into adipocytes and osteoblasts in vitro. (B) Flow cytometric analysis of MSCs surface markers. (C) Morphological features of DFC-sEV were observed via transmission electron microscopy, scale bar = 200 nm. (D) DFC-sEV size and concentration were monitored with NTA. (E) Western blot analysis shows the expression of the representative markers CD9, CD81, HSP90, and TSG101. [file 6625123.f1.docx]
